# The Carriage Of Multiresistant Bacteria After Travel (COMBAT) prospective cohort study: methodology and design

**DOI:** 10.1186/1471-2458-14-410

**Published:** 2014-04-28

**Authors:** Maris S Arcilla, Jarne M van Hattem, Martin CJ Bootsma, Perry J van Genderen, Abraham Goorhuis, Constance Schultsz, Ellen E Stobberingh, Henri A Verbrugh, Menno D de Jong, Damian C Melles, John Penders

**Affiliations:** 1 Department of Medical Microbiology and Infectious Diseases, Erasmus University Medical Center, Wytemaweg 80, 3015 CN Rotterdam, the Netherlands; 2 Academic Medical Center, Department of Medical Microbiology, University of Amsterdam, Meibergdreef 9, 1105 AZ Amsterdam, the Netherlands; 3 Julius Center for Health Research& Primary Care, University Medical Center Utrecht, Heidelberglaan 100, PO 85500, 3508 GA Utrecht, the Netherlands; 4 Department of Mathematics, Faculty of Science, Utrecht University, Budapestlaan 6, PO 80010, 3584 CD Utrecht, the Netherlands; 5 Department of Internal Medicine, Havenziekenhuis - Institute for Tropical Diseases, Haringvliet 2, 3011 TD Rotterdam, the Netherlands; 6 Department of Medical Microbiology, School for Nutrition, Toxicology and Metabolism, Maastricht University Medical Center, PO 5800, 6202 AZ Maastricht, the Netherlands; 7 Department of Epidemiology, School for Public Health and Primary Care, Maastricht University, PO 616, 6200 MD Maastricht, the Netherlands

**Keywords:** Antimicrobial resistance, Extended-spectrum beta-lactamase, Carbapenemase, Enterobacteriaceae, Prospective cohort, Travel

## Abstract

**Background:**

Antimicrobial resistance (AMR) is one of the major threats to public health around the world. Besides the intense use and misuse of antimicrobial agents as the major force behind the increase in antimicrobial resistance, the exponential increase of international travel may also substantially contribute to the emergence and spread of AMR. However, knowledge on the extent to which international travel contributes to this is still limited. The Carriage Of Multiresistant Bacteria After Travel (COMBAT) study aims to 1. determine the acquisition rate of multiresistant Enterobacteriaceae during foreign travel 2. ascertain the duration of carriage of these micro-organisms 3. determine the transmission rate within households 4. identify risk factors for acquisition, persistence of carriage and transmission of multiresistant Enterobacteriaceae.

**Methods/design:**

The COMBAT-study is a large-scale multicenter longitudinal cohort study among travellers (n = 2001) and their non-travelling household members (n = 215). Faecal samples are collected before and immediately after travel and 1 month after return from all participants. Follow-up faecal samples are collected 3, 6 and 12 months after return from travellers (and their non-travelling household members) who acquired multiresistant Enterobacteriaceae. Questionnaires are collected from all participants at each time-point. Faecal samples are screened phenotypically for the presence of extended-spectrum beta-lactamase (ESBL) or carbapenemase-producing Enterobacteriaceae. Positive post-travel isolates from travellers with negative pre-travel samples are genotypically analysed for ESBL and carbapenemase genes with microarray and gene sequencing.

**Discussion:**

The design and scale of the COMBAT-study will enable us to provide much needed detailed insights into the risks and dynamics of introduction and spread of ESBL- and carbapenemase-producing Enterobacteriaceae by healthy travellers and the potential need and measures to monitor or manage these risks.

**Trial registration:**

The study is registered at clinicaltrials.gov under accession number NCT01676974.

## Background

The problem of antimicrobial resistance (AMR) is worldwide one of the foremost health issues that we face in the coming decades [1]. Bacterial AMR reduces clinical efficacy and increases treatment costs. Furthermore, AMR jeopardizes the achievements of modern medicine, since the success of interventions such as organ transplantation, cancer chemotherapy and major surgery depends on effective antimicrobial agents for prevention and treatment of infections. With a dearth of novel antibiotics in the pipeline, the conservation of existing ones is imperative [2].

Next to the well-established role of (inappropriate) antimicrobial use in humans and animals, the exponential increase of international travel may substantially contribute to the emergence and spread of AMR since it allows resistant bacteria or bacterial mobile gene elements carrying resistance genes (e.g. plasmids) to be rapidly transported between regions [3]. To what extent foreign travel poses a risk for the acquisition of AMR remains, however, largely unknown, as the presence of resistant bacteria in the normal human microbiota following travel usually remains undetected unless they cause manifest infection and disease. Yet, due to the high likelihood of contact and genetic exchange with potential pathogens, the human microbiota warrants special attention as perhaps the most accessible reservoir of resistance genes.

Besides being part of the normal human microbiota, Enterobacteriaceae are also important causes of community-acquired and nosocomial infections. Enterobacteriaceae can acquire resistance genes through horizontal gene transfer. Genes encoding for resistance to different classes of antibiotics, such as beta-lactams, quinolones and aminoglycosides are often located on plasmids. Multiple genes, each encoding for resistance to different classes of antibiotics, can be found on the same plasmid [4]. Selective pressure of one antibiotic can therefore lead to resistance to several classes of antibiotics.

Plasmid borne resistance to beta-lactam antibiotics in Enterobacteriaceae is emerging worldwide, due to the production of enzymes called extended-spectrum beta-lactamases (ESBLs). ESBLs have broad-spectrum activity against penicillins, cephalosporins and monobactams by hydrolyzing the beta-lactam ring of these antibiotics, leading to inactivation. Even more worrisome, Enterobacteriaceae can acquire resistance genes encoding for enzymes called carbapenemases. These carbapenemase-producing Enterobacteriaceae (CPE’s) are “extreme drug resistant”. Their enzymes are active against our last resort class of antibiotics: the carbapenems. Up to now, only case-reports have shown acquisition or infection with CPE’s among travellers upon visit or hospitalization in endemic areas [5].

Besides horizontal gene transfer, AMR bacteria can spread from the traveller to other family members and beyond, through the faeco-oral route [6]. The traveller can therefore be seen as an interactive biological unit who picks up, processes, carries and drops off microbial genetic material [7]. Consequently, local emergence of AMR can rapidly result in worldwide spread.

So far, five small to medium-sized prospective studies (n = 40–370) have investigated acquisition of AMR Enterobacteriaceae during international travel. These studies reported acquisition rates of ESBL-producing Enterobacteriaceae (ESBL-E) in faeces ranging from 24% to 33% among Swedish, Australian, American and Dutch travellers, with acquisition rates up to 88% depending on destination [8-12]. No acquisition of carbapenemase-producing Enterobacteriaceae was found.

In an earlier prospective study among travellers, a rapid decline in carriage of resistant isolates was demonstrated. A relative small proportion (10%) of subjects had persistent carriage after 6 months [13].

While these studies identified international travel as an important risk factor for acquiring AMR microorganisms, several important questions still need to be fully addressed to understand the contribution of travel to AMR emergence and spread, to assess the risk for public health and to identify measures to manage this risk. These knowledge gaps include (1) identification of travel-associated risk factors (including destination) for acquisition and subsequent carriage of these resistant microorganisms, (2) duration of colonization with AMR strains acquired during travel, (3) probability and dynamics of subsequent transmission of AMR strains within households and (4) the proportion of colonized travellers who develop infections with these resistant bacteria.

### Scope of research

The Carriage Of Multiresistant Bacteria After Travel (COMBAT) Study, aims to prospectively study the influence of international travel and travel-associated risk factors on the acquisition, persistence and transmission of AMR in the endogenous microbiota of healthy individuals. The specific aims are:

1. to determine the acquisition rate of ESBL- and carbapenemase-producing Enterobacteriaceae during foreign travel by comparing pre- and post-travel faecal samples;

2. to ascertain the duration of carriage of these microorganisms (or their resistance genes/mobile genetic elements) by studying faecal specimens at regular intervals up to 1 year after return;

3. to mathematically model the decolonization and transmission rates of these imported Enterobacteriaceae (or their resistance genes/mobile elements) within households by prospectively studying consecutive specimens from household members (who did not join the index case on his/her travel);

4. to identify the risk factors for acquisition, persistence of carriage and transmission of ESBL- and carbapenemase-producing Enterobacteriaceae;

5. to examine whether carriers of resistant Enterobacteriaceae have a higher risk of bacterial infections in the year after travel (compared to non-carriers).

## Methods/design

### Design

**Figure 1:**
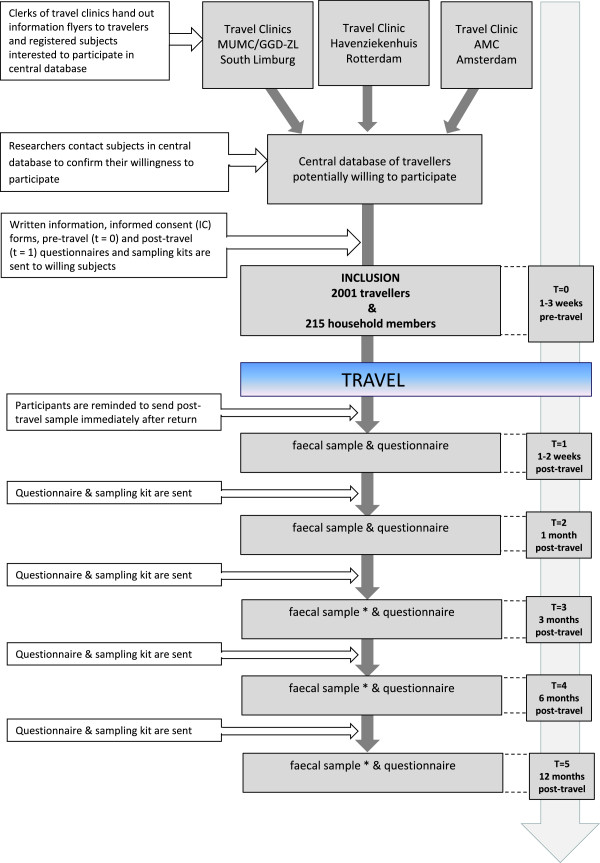
**Flowchart of study design.** * Depending on colonization status of traveller (or his/her household member) at previous time-points.

The design of the COMBAT-study is a multicenter longitudinal cohort study among travellers who are followed from one week prior to travel departure until 12 months after return. In order to study household transmission, non-travelling household members are also included and are followed over the same period as their travelling household members. Figure 1 depicts a flowchart of the study design and procedures.

### Study area and recruiting centers

Participants are recruited at the outpatient clinics run by the Academic Medical Center (Amsterdam, the Netherlands), Havenziekenhuis (Rotterdam, the Netherlands) and Maastricht University Medical Center/Public Health Service South Limburg (Maastricht, the Netherlands), which together are visited by approximately 52.700 travellers each year for travel advice and vaccinations. Subjects are recruited within a period of one year, from November 2012 until November 2013.

### Eligibility criteria

Travellers - Eligible subjects are adult (≥18 years) volunteers visiting one of the above stated travel clinics, travelling abroad for a minimum of one week to a maximum of three months. Minors (<18 years) and incapacitated subjects are excluded from this study.

Non-travelling household members–Non-travelling adult household members of participating travellers are also enrolled. A household contact is defined as an individual who lives in the same house as the traveller and shares the same kitchen and/or bathroom and/or toilet on a regular basis.

### Sample size and power calculation

In order to determine the minimum number of travellers required to detect risk factors for acquisition of ESBL-producing Enterobacteriaceae with sufficient power, the following assumptions were made: 1. a 2% pre-travel prevalence of carriage of ESBL-producing Enterobacteriaceae; 2. an acquisition rate of ESBL-producing Enterobacteriaceae of 24% during travel; 3. a two-sided significance level (alpha) of 0.05; 4. a power (1-beta) of 80%; 5. a minimum odds ratio of 2.0; 6. a minimum prevalence of a travel-associated risk factor of 5%. Based upon these assumptions a sample size of 1541 analyzable subjects is required. Accounting for an estimated attrition rate of 20% immediately after travel (t = 1), a total of 1926 travellers need to be recruited.

After one year of follow-up, 2001 travellers were included fulfilling the requested sample size. To minimize the drop-out and non-response levels, participants are reminded through several channels in case questionnaires or samples are not received in time. Participants are sent reminders initially by emails, followed by text messages to their mobile phones and, in case of no responses are received, are finally contacted by telephone by one of the researchers. This resulted in an attrition rate immediately after travel (t = 1) of 1,6%, being far lower than expected. Table 1 shows minimal effect sizes that can be detected within the final cohort according to a prevalence of a risk factor ranging from 5 to 50%.

**Table 1:** Effect sizes that minimally can be detected according to the prevalence of the exposure in the final cohort of 2001 travellers

**Proportion exposed (%)**	**Odds ratio**
50%	1.36
25%	1.41
10%	1.64
5%	1.92

### Study procedures

#### Eligibility screening activities

All clerks of the participating outpatient travel clinics are instructed to hand out an information flyer on the COMBAT-study to all travellers visiting the clinics during the recruitment period. If travellers are interested to participate they are instructed to fill in the flyer with their contact details, date of departure and return, and number of non-travelling household members. Travellers who meet the eligible criteria are provided with additional information on the study procedure and subsequently contacted by phone to confirm their willingness to participate. Travellers not fulfilling the eligible criteria receive an email informing them on the reason for exclusion.

#### Ethical approval and informed consent

Subjects willing to participate are subsequently sent written information on the study procedures along with an informed consent form. Only participants providing written informed consent are enrolled. Ethical approval was obtained by the Medical Ethical Committee of Maastricht University Medical Center (study number: METC 12-4-093).

### Data collection

#### Faecal sampling

Travellers and if applicable their participating non-travelling household members are instructed to self-collect a faecal sample before travel (t = 0) as well as immediately (t = 1) and one month (t = 2) after return. In case any of these samples from a traveller or his/her household member(s) is positive for ESBL- or carbapenemase-producing Enterobacteriaceae, both the traveller and the household member(s) are asked to provide additional samples at each subsequent follow-up moment (3, 6 and 12 months after travel, t = 3-5). Sample collection and shipment kits are sent to participants before travel (for the collection of samples at t = 0 and t = 1), 2 weeks prior to the subsequent follow-up timepoint (t = 2) and if applicable 2 weeks prior to each of the follow-up time points (t = 3-5). A sample collection and shipment kit consists of an instruction form, a safety bag, a bibulous tissue, a postage paid airbag envelope and a faeces collection swab with modified Cary Blair transport medium (Fecal Swab®; Copan, Brescia, Italy). Participants are instructed to sample fresh stools by turning the swab into faeces without touching the toilet or water, package the sample according to the instructions and send to the laboratory immediately.

At the laboratory, samples are processed upon arrival. Residuals are aliquoted and stored at −80°C for future research.

### Questionnaires

Questionnaires (in Dutch language) are sent to all participants at each timepoint. (t = 0-5). All questionnaires collect information on the date of sample collection and gastro-intestinal symptoms, including the ROME III IBS diagnostic questionnaire [14]. The pre-travel questionnaire (t = 0) comprises detailed information on demographic parameters (e.g. ethnicity, gender, age, household composition), travel history in the past years, pre-existing morbidity and medication use, hospital admissions and antibiotic use during the past year, as well as dietary preferences. The first post-travel questionnaire (t = 1) mainly collects information on travel details, such as duration; destination(s); urban/rural travel; type of travel (e.g. business, family visit, holiday); lodging (e.g. hotel, tent, family, locals); ailments or illnesses during travel (i.e. gastroenteritis); hospital admission; medical interventions and use of medication (in particular antibiotic use); place of meal consumption (e.g. at hotel, local restaurants, food stalls); unboiled/unbottled water consumption. The questionnaires at each subsequent follow-up collect data on intercurrent travel, medication use (including antibiotic use), hospital admissions and occurrence of illnesses/infections.

If applicable, travellers are asked to provide data on their relationship to the household members who also participate in the study. This includes data on the type of relationship (roommate, partner, parent, child, sibling, other), forms of contact (e.g. sharing of bed, towel, toothbrush, balms/lotions) and on household characteristics (e.g. household size). Participating non-travelling household members also receive questionnaires at each timepoint (t = 0-5) on demographic parameters, travel history, travel during the study period, medication use, hospital admission and occurrences of illness/infections.

### Microbiological methods

#### Bacterial culture and antibiotic susceptibility testing

Faecal samples are processed immediately upon arrival at the laboratory. The samples are selectively enriched: 100 microliter of the liquid medium with faeces is pipetted into 5 ml of tryptic soy broth (TSB) supplemented with vancomycine (50 mg/l), followed by overnight incubation at 35°C [15]. The next day, volumes of 10 microliters are inoculated on chromID**®** ESBL (bioMérieux, Marcy l’Etoile, France), a selective agar plate to screen for ESBL- and carbapenemase-producing Enterobacteriaceae. These agar plates are incubated overnight at 35°C. All colonies growing on chromID® ESBL agar are further characterized to the species level using MALDI-TOF (Bruker, London, United Kingdom). Minimum inhibitory concentrations (MIC) are measured for all Enterobacteriaceae by the use of the automated susceptibility testing system Vitek 2 (bioMérieux, Marcy l’Etoile, France). The susceptibility testing results are interpreted by the clinical breakpoints recommended by EUCAST (the European Committee on Antimicrobial Susceptibility Testing). Phenotypic confirmation of ESBL-producing Enterobacteriaceae is performed by the combination disk diffusion test according to current national Dutch guidelines. Enterobacteriaceae with an MIC for imipenem and/or meropenem above the recommended screening breakpoint(s) measured by the Vitek 2 will be confirmed by Etest (bioMérieux, Marcy l’Etoile, France) [16].

#### Genotypic characterization

ESBL- and carbapenemase-producing post-travel isolates (t = 1) from travellers with negative pre-travel samples (t = 0) are screened for the presence of multiple classes of ESBL and carbapenemase genes using microarray (Identibac® AMR08; Alere Technologies GmbH, Jena, Germany). This platform is a miniaturized DNA-hybridization array in a strip based system for the detection of >120 antimicrobial resistance genes in Gram-negative bacteria, including those conferring resistance to aminoglycosides, trimethoprim, sulphonamides, tetracyclines, quinolones, and beta-lactams, including ESBLs and carbapenemases. In case of positive microarray signals, targeted PCR and DNA sequencing will be used to further genetically characterize the specific type of ESBL or carbapenemase in t = 1 isolates. DNA sequences will be analyzed using existing DNA databases (NCBI GenBank and Lahey beta-lactamase classification and amino acid sequences for TEM, SHV and OXA-Extended-Spectrum and Inhibitor Resistant Enzymes) which are updated regularly.

In case of negative microarray results of phenotypically resistant isolates, additional screening will be performed by PCR. To confirm persistence of colonization and/or transmission, phenotypically confirmed ESBL- or carbapenemase-producing isolates from follow-up samples of travellers and household members will be tested by targeted PCR and DNA sequencing (based on results from t = 1). Clonal bacterial spread within households will be confirmed or excluded by molecular (plasmid-) typing.

## Results

**Figure 2:**
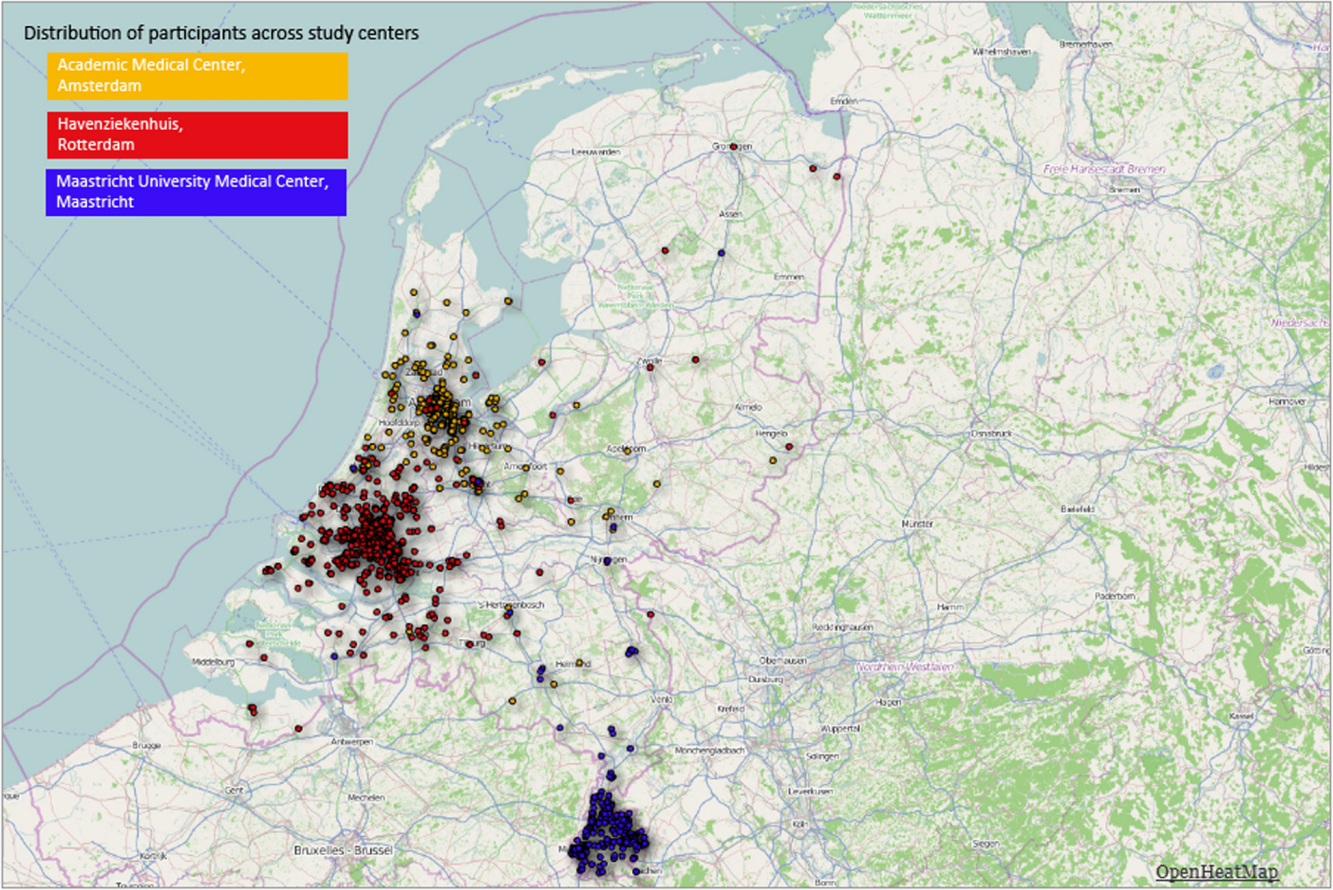
**Geographic distribution of residences of participating travellers (n = 2001) throughout the Netherlands according to study center.** i. Yellow circles represent participants from Tropencentrum AMC, Amsterdam. ii. Red circles represent participants from Travel Clinic Havenziekenhuis, Rotterdam. iii. Blue circles represent participants from Maastricht University Medical Center, Maastricht.

**Figure 3:**
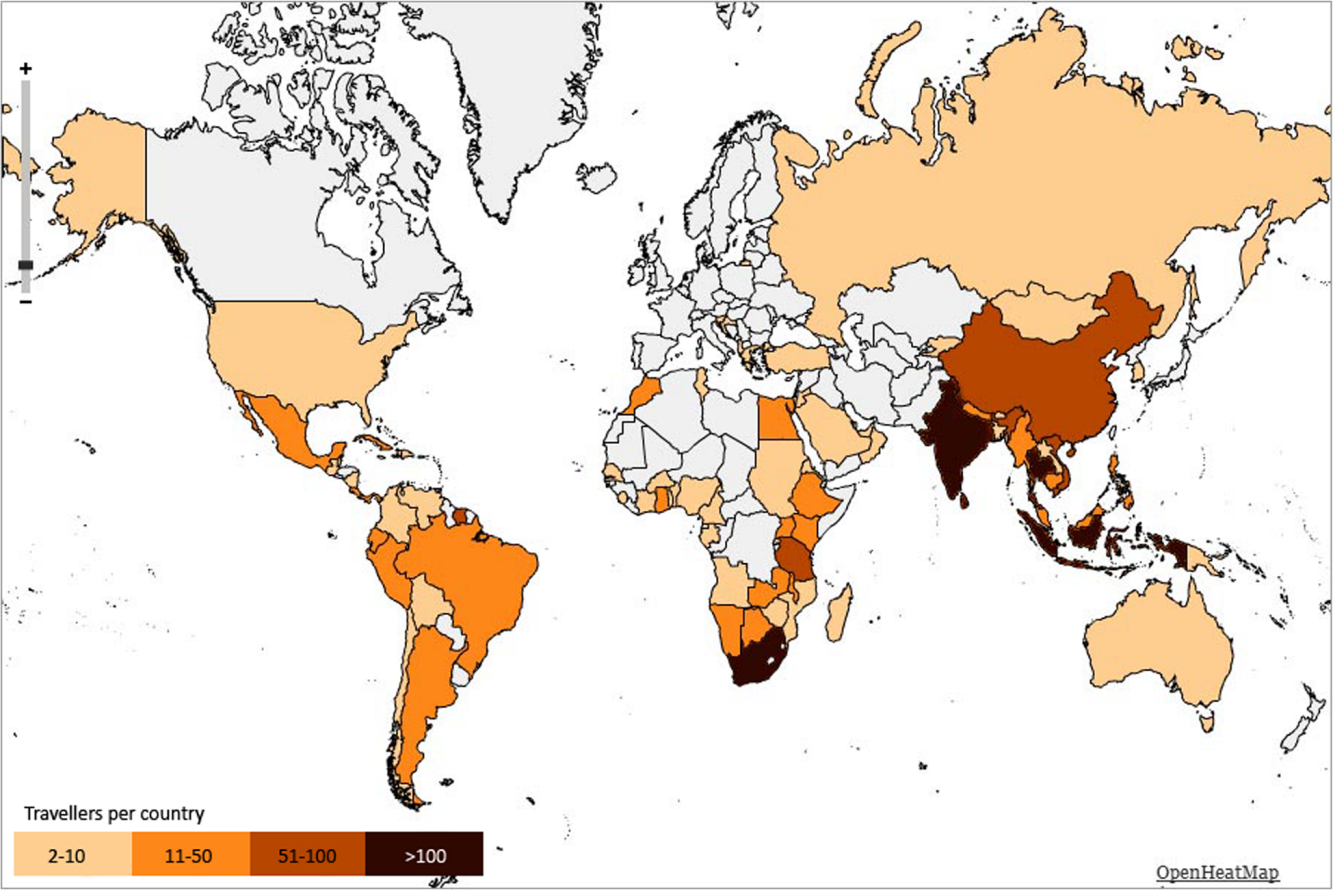
**Heatmap showing the countries visited by the participating travellers (n = 2001).** i. Grey color indicates 0–1 travellers visited country. ii. Light yellow color indicates 2–10 travellers visited country. iii. Orange color indicates 11–51 travellers visited country. iv. Light brown color indicates 50–100 travellers visited country. v. Dark brown color indicates > 100 travellers visited country.

2001 travellers and 215 non-travelling household members were included. The median age of travellers and household members is respectively 50.5 years (range 18.1-81.7) and 46.9 years (range 18.4-82.0), 54.0% of travellers and 62.8% of household members are female (center-specific characteristics are presented in Table 2). The distribution of the participants throughout the Netherlands and across study centers is depicted in Figure 2. The regions most frequently visited were South-Eastern Asia, Eastern Africa, Southern Asia and South America (Figure 3).

**Table 2:** Baseline characteristics of travellers and non-travelling household members according to study center

	**Rotterdam**		**Amsterdam**		**Maastricht**		**Total**	
	Travellers	Household members	Travellers	Household members	Travellers	Household members	Travellers	Household members
	(n = 1110)	(n = 129)	(n = 496)	(n = 43)	(n = 395)	(n = 43)	(n = 2001)	(n = 215)
**Sex**								
Male	541 (48.7%)	39 (30.2%)	208 (41.9%)	18 (41.9%)	171 (43.3%)	23 (53.5%)	920 (46.0%)	80 (37.2%)
Female	569 (51.3%)	90 (69.8%)	288 (58.1%)	25 (58.1%)	224 (56.7%)	20 (46.5%)	1081 (54.0%)	135 (62.8%)
**Age in years** (median, range)	52.0 (18.1-81.7)	46.3 (18.4-82.0)	44.7 (19.8-74.6)	41.1 (18.9-78.0)	50.4 (18.2-71.9)	50.6 (18.4-71.6)	50.5 (18.1-81.7)	46.9 (18.4-82.0)
**Continents visited by traveller**								
Asia	557 (50.2%)		259 (52.2%)		200 (50.6%)		1016 (50.8%)	
Africa	362 (32.6%)		148 (29.8%)		123 (31.1%)		633 (31.6%)	
America	177 (15.9%)		81 (16.3%)		68 (17.2%)		326 (16.3%)	
Europe	11 (1.0%)		6 (1.2%)		4 (1.0%)		21 (1.0%)	
Oceania	3 (0.3%)		2 (0.4%)		0 (0.0%)		5 (0.2%)	

## Discussion

The design and scale of the COMBAT-study are optimal to study the influence of international travel and travel-associated risk factors on the acquisition, persistence of carriage and transmission of AMR Enterobacteriaceae. A limited number of previous studies have suggested high acquisition rates of AMR Enterobacteriaceae during international travel, but most did not examine the duration of colonization and none looked at local transmission of imported AMR. Our larger scale longitudinal studies will not only assess the probability of colonization by AMR Enterobacteriaceae during international travel along with associated risk factors, but will also determine the duration of such colonization as well as the probability and dynamics of subsequent transmission of AMR within households. In addition, while the main focus of the project will be on ESBL- and carbapenemase-producing Enterobacteriaceae, innovative molecular approaches (microarray) will be used to provide a more comprehensive and complete picture of associated resistance genes acquired during travel. Our extensive data from questionnaires will identify travel-associated risk factors for acquisition, persistence and transmission of AMR Enterobacteriaceae.

Selection towards a more affluent and healthy study population is a common phenomenon in epidemiological studies and has likely also occurred in our study. This potential selection may be related to some determinants and outcomes separately (non-differential selection), affecting the frequency rates and, as a consequence, the statistical power and generalizability of the results [17]. However, since we have access to the demographic data of all visitors of the travel clinics during the recruitment period, we will be able to perform detailed non-response analysis and examine to what extent the study population deviates from its source population. The incidence rates of AMR acquisition found in the study will be interpreted accordingly. Moreover, this selection would only lead to bias in etiological association studies if the selection mechanisms are related to both the determinant and the outcome (differential selection), which is, in contrast to retrospective and cross-sectional studies, unlikely in the present prospective study.

Major efforts have been made to keep the follow-up rates as high as possible and to prevent (selective) loss to follow-up. This has resulted in follow-up rates as high as 98.4% immediately after travel (t = 1). Taken together, results from this study will provide much needed detailed insights into the risks and dynamics of introduction and spread of AMR by healthy travellers and the potential need and measures to monitor or manage these risks.

## Competing interests

The authors declare they have no competing interests.

## Authors’ contributions

MSA and JMH conduct the study and contributed to the design. MSA and JP drafted the manuscript. MDdJ, CS, DCM, HAV, JP designed the study and they are members of the supervising board. MCJB, PJG, EES, AG were involved in the design of the study. MSA, JMH, and JP revised several early drafts of the paper and MDdJ and DM commented on the final draft. All authors read and approved the final manuscript.

## Pre-publication history

The pre-publication history for this paper can be accessed here:

http://www.biomedcentral.com/1471-2458/14/410/prepub
